# Woman With Shoulder Pain: Posterior Shoulder Dislocation Diagnosed With Point-of-Care Ultrasound

**DOI:** 10.7759/cureus.64180

**Published:** 2024-07-09

**Authors:** Steven Gayda, Brian Kohen, Eric Boccio

**Affiliations:** 1 Emergency Medicine, Memorial Healthcare System, Pembroke Pines, USA

**Keywords:** pocus (point of care ultrasound), musculoskeletal pain, shoulder pain, point-of-care ultrasound (pocus), shoulder dislocation

## Abstract

Posterior shoulder dislocations are relatively rare. When used by emergency medicine physicians, point-of-care ultrasound (POCUS) demonstrates higher sensitivity and specificity for diagnosing shoulder dislocation as compared to two-view plain films.

A 49-year-old woman presented to the emergency department (ED) with left shoulder pain following a mechanical fall. Physical examination was remarkable for a gross shoulder deformity and tenderness over the left proximal humerus. POCUS of the left shoulder using a curvilinear probe and a posterior approach was performed and demonstrated posterior displacement of the humeral head relative to the glenoid. Anteroposterior and oblique shoulder X-rays were read as unremarkable by the radiologist; a computed tomography of the shoulder confirmed a posterior shoulder dislocation.

Given its efficacy and efficiency as compared to X-ray radiography, POCUS should be strongly considered in the diagnosis and management of posterior shoulder dislocations in the ED setting.

## Introduction

Shoulder dislocations are a common cause of presentation to the emergency department (ED). While radiographs remain the standard imaging modality for identifying and guiding reduction of shoulder dislocations, the diagnostic utility of ultrasonography warrants exploration. This case illustrates the utility of point-of-care ultrasound (POCUS) in diagnosing a posterior shoulder dislocation despite normal shoulder radiographs.

## Case presentation

**Figure 1:**
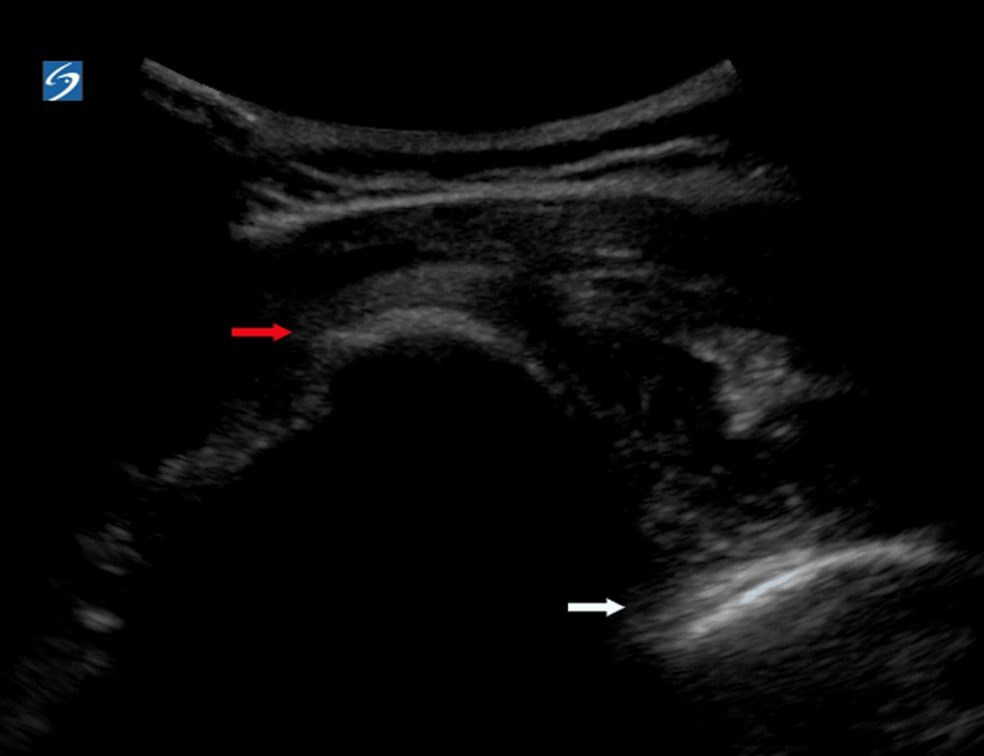
Pre-reduction point-of-care ultrasound of the left shoulder using a curvilinear probe and a posterior approach demonstrating posterior displacement of the humeral head (red arrow) relative to the glenoid rim (white arrow)

A 49-year-old woman with a noncontributory past medical history presented to the ED with acute left shoulder pain following a mechanical fall. The patient denied other injuries and complaints. Physical examination was remarkable for a gross deformity and tenderness over the left proximal humerus and shoulder, strong radial pulse, and intact sensation along the left upper extremity. The patient was unable to move the extremity due to pain. Anteroposterior (AP) and oblique shoulder X-rays were ordered, and the patient was given intravenous morphine. While awaiting plain films, bedside POCUS of the left shoulder using a curvilinear probe with a posterior approach was performed. POCUS demonstrated posterior displacement of the humeral head relative to the glenoid, raising suspicion for a posterior shoulder dislocation (Figure 1).

**Figure 2:**
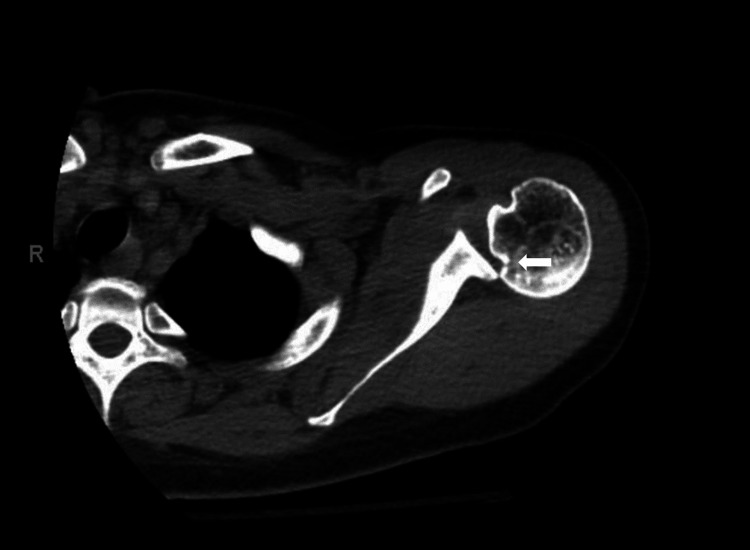
Computed tomography of the left shoulder demonstrating posterior shoulder dislocation and reverse Hill-Sachs deformity (white arrow)

AP and oblique shoulder X-rays were read as normal with no evidence of fracture, dislocation, or subluxation by the radiologist. Interpretation was noted to be limited due to suboptimal patient positioning. Due to the high clinical suspicion of posterior shoulder dislocation based on physical examination and ultrasound findings, computed tomography (CT) of the left shoulder was obtained. CT imaging confirmed a posterior shoulder dislocation with reverse Hill-Sachs deformity (Figure 2).

A closed reduction was performed under conscious sedation using fentanyl and ketamine. Post-reduction X-rays demonstrated satisfactory positioning. The patient tolerated the procedure well with no complications, was placed in a shoulder sling, and discharged home with outpatient orthopedic surgery follow-up.

## Discussion

Although shoulder dislocations are a common cause of presentation to the ED, posterior shoulder dislocations are relatively rare, occurring in only 2-4% of all cases [1]. Posterior shoulder dislocations may be challenging for the emergency medicine (EM) physician to diagnose due to subtleties related to the review of systems, physical examination, and radiographic findings [2]. Typical mechanisms leading to posterior shoulder dislocation include axial loading of an adducted and internally rotated upper extremity from trauma and rigorous muscle contractions associated with seizures and electrocution [3]. While three-view radiographs remain the standard imaging modality for diagnosing and confirming the successful reduction of shoulder dislocations, ultrasonography is becoming increasingly popular given its advantages [4]. POCUS can be used to diagnose, guide intra-articular anesthetic injection and nerve blocks, and confirm relocation following reduction attempts at the bedside while the patient is still sedated [5-6]. Additionally, POCUS images can be obtained more rapidly than radiographs allowing for faster identification, decreased lengths of stay, and more efficient ED throughput [7]. Furthermore, POCUS may obviate the need for patient transfers to radiology suites, decrease radiation exposure, and reduce healthcare-related costs [8]. The use of POCUS in the diagnosis and management of shoulder dislocations can be learned and mastered by inexperienced operators quickly and with high success. When used by EM physicians, POCUS demonstrated >99% sensitivity and >99% specificity in identifying shoulder dislocations and reductions when compared to reference standards [9]. The sensitivity and specificity of POCUS in diagnosing non-Hill-Sachs/Bankart’s humeral fractures are also quite high, 92% and 100%, respectively [7]. A systematic review and meta-analysis illustrated that the posterior approach had greater diagnostic accuracy than the anterior/lateral technique with no significant differences seen between attempts utilizing the curvilinear versus linear probe [10].

## Conclusions

Often missed on plain imaging, POCUS demonstrates high sensitivity and specificity in diagnosing posterior shoulder dislocations. POCUS may offer advantages when compared to standard radiographic imaging, including reduced radiation exposure, more expeditious usage at the bedside, and real-time guidance of nerve blocks and reduction attempts. When combined with a comprehensive medical history and physical examination, POCUS is a useful tool when diagnosing shoulder dislocations in the ED setting.
